# Retraction Note: SP1-induced lncRNA AGAP2-AS1 expression promotes chemoresistance of breast cancer by epigenetic regulation of MyD88

**DOI:** 10.1186/s13046-023-02596-2

**Published:** 2023-01-11

**Authors:** Huaying Dong, Wei Wang, Shaowei Mo, Ru Chen, Kejian Zou, Jing Han, Fan Zhang, Jianguo Hu

**Affiliations:** 1 Department of General Surgery, Hainan General Hospital, Jinan University, No.19 Xiu Hua Road, Xiuying District, Haikou city, 570311 Hainan Province China; 2 Department of Science and Education, Hainan Maternal and Child health hospital, Haikou, 570206 Hainan China; 3 Department of Obstetrics and Gynecology, The Second Affiliated Hospital, Chongqing Medical University, Chongqing, 400010 China

**Retraction Note:** ***J Exp Clin Cancer Res*** **37, 202 (2018)**

**https://doi.org/10.1186/s13046-018-0875-3**

The Editor-in-Chief has retracted this article because data presented in Figs. 2, 3 and 4 were duplicated from previously published articles. Specifically, a set of staining images in Fig. 2I had previously been published as Fig. 3E in [1]. The actin blot in Fig. 3D had been published as part of Fig. 4B in [2], and as part of 7H in [3]. The BT474 blot in Fig. 4C was previously published as P2 in Fig. 2F of [4]. The SKBR-3 MyD88 blot in Fig. 4D originally appeared as snail in Fig. 5D in [5]. The Editor-in-Chief no longer has confidence in the results and conclusions of this article. Huaying Dong states that all authors agree to this retraction.
